# CIAO: a living experiment in interdisciplinary large-scale collaboration facilitated by the Adverse Outcome Pathway framework

**DOI:** 10.3389/fpubh.2023.1212544

**Published:** 2023-08-10

**Authors:** Annamaria Carusi, Julija Filipovska, Clemens Wittwehr, Laure-Alix Clerbaux

**Affiliations:** ^1^Interchange, London, United Kingdom; ^2^Independent Scientist, Ohrid, North Macedonia; ^3^European Commission, Joint Research Centre (JRC), Joint Research Centre, Ispra, Italy

**Keywords:** interdisciplinarity, collaboration, adverse outcome pathway, crowdsourcing, knowledge management

## Abstract

**Introduction:**

The CIAO project was launched in Spring 2020 to address the need to make sense of the numerous and disparate data available on COVID-19 pathogenesis. Based on a crowdsourcing model of large-scale collaboration, the project has exploited the Adverse Outcome Pathway (AOP) knowledge management framework built to support chemical risk assessment driven by mechanistic understanding of the biological perturbations at the different organizational levels. Hence the AOPs might have real potential to integrate data produced through different approaches and from different disciplines as experienced in the context of COVID-19. In this study, we aim to address the effectiveness of the AOP framework (i) in supporting an *interdisciplinary* collaboration for a viral disease and (ii) in working as the conceptual mediator of a *crowdsourcing* model of collaboration.

**Methods:**

We used a survey disseminated among the CIAO participants, a workshop open to all interested CIAO contributors, a series of interviews with some participants and a self-reflection on the processes.

**Results:**

The project has supported genuine *interdisciplinarity* with exchange of knowledge. The framework provided a common reference point for discussion and collaboration. The diagram used in the AOPs assisted with making explicit what are the different perspectives brought to the knowledge about the pathways. The AOP-Wiki showed up many aspects about its usability for those not already in the world of AOPs. Meanwhile their use in CIAO highlighted needed adaptations. Introduction of new Wiki elements for modulating factors was potentially the most disruptive one. Regarding how well AOPs support a *crowdsourcing* model of large-scale collaboration, the CIAO project showed that this is successful when there is a strong central organizational impetus and when clarity about the terms of the collaboration is brought as early as possible.

**Discussion:**

Extrapolate the successful CIAO approach and related processes to other areas of science where the AOP could foster interdisciplinary and systematic organization of the knowledge is an exciting perspective.

## 1. Introduction

### 1.1. The CIAO project

The challenge of Coronavirus disease 2019 (COVID-19) has absorbed a great deal of attention from scientists and policy makers since its inception. It is a complex disease that is highly heterogeneous in clinical outcomes. Since the onset of the pandemic, scientists worldwide have been engaged in researching the underlying mechanisms of the disease, in a bid to improve preventive measures and treatment options. Scientific publications dealing with this disease have increased exponentially. The data are produced through *in vitro*, *in vivo*, clinical and epidemiological studies as well as *in silico* models. While research tends to be compartmentalized in silos, the pandemic clearly called for an interdisciplinary integration of knowledge and data from the different experimental systems. In Spring 2020, the CIAO [*Modeling the COVID-19 pathogenesis using the Adverse Outcome Pathway framework*] project was launched to address the need for an interdisciplinary forum to combine the numerous and disparate data available on COVID-19 pathogenesis (1). The project exploits the Adverse Outcome Pathways (AOP) framework, an internationally recognized toxicological knowledge framework originally built to support chemical risk assessment based on mechanistic reasoning (2, 3). Steered by the Joint Research Centre Unit for Systems Toxicology of the European Commission, the project was launched through a call for collaboration. Very soon, participants began to join in, and drew in further participants, with 80 researchers joining at different times, and an average of between 40 and 50 active participants over the duration of the project. Participants came from across disciplines, across sectors, and across nations (20 countries in Europe, US and Asia). The project has produced a large number of peer-reviewed publications (4–10), and many COVID-19 related AOPs uploaded on the AOP-Wiki,^1^ to be (re-)used by other researchers, with the aspiration of also being useful for healthcare practitioners, treatment developers, and policy makers.

### 1.2. Background and enabling conditions

From its inception, the CIAO project had a clear scientific aim: organizing knowledge about the pathways of the disease through the AOP framework. This aim could be achieved only through specific conditions that would allow different disciplines to collaborate effectively. We refer to these conditions as broadly social, though they include institutional, organizational, and interpersonal aspects; as well as a large role for social epistemological factors. In this section we outline three different elements of the CIAO project: (i) the AOP framework (the conceptual scientific backbone of the collaboration); (ii) the crowdsourcing approach to the collaboration and (iii) the steered organization of the project.

#### 1.2.1. The AOP framework

The AOP framework facilitates identification, structured presentation, assessment and communication of the knowledge underpinning mechanistic understanding of the biological perturbations at different organizational levels—from the molecular level via cellular, tissue and organ level, up to an adverse outcome at the organism level (2, 3). The AOP framework is steered within the OECD which also maintains an online centralized platform called AOP-Wiki,^2^ where all AOPs developed are openly accessible. AOPs have been mainly explored so far within the field of toxicology for chemical, nanomaterial and radiation safety assessment (11–13). The CIAO project was based on the assumption that the AOP framework could also support systematic organization of the diverse and fast-evolving knowledge on COVID-19 pathogenesis (1, 14). AOPs have indeed real potential to mediate breaking of the silos of knowledge as they integrate data and information produced through different approaches, and from different disciplines. An AOP-aligned identification, curation and integration of relevant data may significantly enhance confidence in existing knowledge, point toward lack of knowledge and guide research to address knowledge gaps (1, 14). This mechanistic organization can also help to capture how factors, such as sex, gender, age, comorbidities, diet or exposure to chemicals, modulate the onset, progression and severity of the disease (4).

#### 1.2.2. Crowdsourcing model of collaboration

Integrating knowledge about COVID-19 requires collaboration across all forms of boundaries: disciplinary, sectors and geographical locations. By its nature, it needs participants who are willing to pool their specialist knowledge and actively collaborate with others to produce AOPs relevant to COVID-19 as collective products. The project was established using a crowdsourcing approach. This is defined in a wide variety of ways (15). In general, crowdsourcing can be used to gather together all kinds of contributions by people of different levels of expertise, and often but not always remunerates member of the crows financially, or offers some other form of recompense. In the case of CIAO, the call to any potential interested members as individuals was initiated by the Systems Toxicology Unit of the European Commission’s Joint Research Centre. The outreach to parties potentially interested in becoming part of the CIAO project was almost exclusively based on individuals communicating among each other (e-mails to acquaintances, sending out and reaction to social media posts from familiar actors, falling back to one’s networks, word of mouth…). As with similar crowdsourcing calls, potential participants are appealed to on the basis of their own values and interests, with the understanding that there will be mutual benefit, even though not everyone necessarily gets the same benefits (16). The size of the potential membership is defined by what is being contributed and which resources are required. In crowdsourcing for scientific purposes the task is highly specialized and those eligible to contribute constitute a fairly restricted community of researchers (17, 18). Yet, it is still a dispersed community, in that it is international and interdisciplinary (toxicologists, chemists, biologists, clinicians, and related expertise) and cross-sectoral (academics, industry researchers, governmental and NGOs researchers). Even though the call may come from a central agency or organization, the group coming together in this collaboration (the “crowd” in crowdsourcing terminology) is by no means passive; but is comprised of people with needs and values which must be understood and met if the process is to be successful. The motivations of people to get involved with what can be time-consuming collaborations, and often altruistic and not necessarily work-related contributions are highly complex, and require as much attention as the integration of knowledge.

There were two dominant motivations behind the establishment of the CIAO project: (i) the desire to contribute something useful to the scientific community faced with the challenge of this deadly disease with widespread health, social, economic and political consequences; (ii) the goal of promoting and developing the AOP framework itself.

#### 1.2.3. Organization

The project had a clear organizational center in the JRC Unit for Systems Toxicology, which saw it fulfilling the dual roles of extending the use of the AOP framework (which the Unit has been instrumental in developing and promoting), and making a contribution to the efforts to gather knowledge of COVID-19. By Autumn 2020, the project gathered more than 40 participants and had initiated sufficient discussions for different working groups focusing on specific organs, or aspects of the disease (4, 14). The working groups were steered by different coordinators. The existence of different working groups called for a central steering or coordination group to be formed, to maintain an overview of the topics to be considered, as these emerged and co-evolved with the project, look for cross-cutting opportunities between the working groups, and keep a checklist of the tasks to be performed. The need for strong coordination became more pressing as the project increased in its scope and in the number of participants, a full-time project coordinator was appointed. The project also had a dedicated communicator, who produced regular newsletters (30 in total), keeping everyone from the project up to date with the activities of all the other working groups. The coordination group (composed of the main project coordinator, the different working group coordinators and the communicator notably) met twice monthly. The working groups established their own schedules, ranging from short meetings weekly, to longer meetings every 6 weeks. For each meeting, there was an agenda circulated in advance, and minutes circulated afterwards. The main collaborating online platform within CIAO was the AOP-Wiki, although other social (Slack) and scientific information management (Zotero) tools and platforms (Google drive, Share Point) were also used transiently.

## 2. Research questions

The project included a “meta-level” reflection on its own practices from about mid-way through. The developers of the AOP approach have an abiding interest in the uptake of the framework, and have been aware of the social dimension of the framework (19). The term “social” can include a broad array of factors. In the case of this project, a first main focus was on understanding the mediation of interdisciplinary collaboration through structures similar to the AOP framework, informed by a broad literature on scientific tools as mediators [a classic being (20)]; with an application to biosciences in (21). The second main focus was the role such frameworks can have in the nature of the inter-relationships among participants contributing via a crowdsourcing model: their motivations, their rewards, and the unfolding of the collaboration over disagreements as well as agreements. Here we will address the following two research questions.

### 2.1. How effective is the AOP framework in supporting an *interdisciplinary collaboration* in the context of a viral disease?

From the outset, the AOP framework aimed to facilitate gathering and organizing data and information from across disciplines. Interdisciplinarity is “hard baked” into it, as it seeks to combine information about a pathway across different levels of biological organization, which by the nature of the case, involves different methods, approaches and disciplines. This is the case, even when the AOP framework is used in its original area of toxicology, which is a multi-disciplinary and multi-approach area of study. However, in extending the framework to viral diseases, participants were dealing with two interconnected aspects at the same time: their understanding of each other, and their understanding of each other from their different disciplinary perspectives. This means that the effectiveness of the AOP framework in supporting interdisciplinarity is closely intertwined with its effectiveness as a framework that can be used successfully for disease pathways. As a scientific question this is addressed elsewhere. The connection between these two aspects is discussed further in section 4.2.

### 2.2. How effective is the AOP framework as a conceptual mediator for a c*rowdsourcing model of collaboration*?

The AOP framework’s success depends on generating input from many different users, who will develop, upload and use AOPs, discover opportunities for collaboration to enrich different parts of AOPs with data and evidence or to build AOPs that become networks. Encouraging collaboration has been its ethos from the outset, with the AOP-Wiki as the collaborative platform that is essential to the AOP vision. This vision of AOPs means that it has always had a dual scientific and social role: both as a system for gathering, organizing and synthesizing knowledge; and as a necessarily collaborative effort. From the outset, it has used a crowdsourcing model of collaboration, with the Wiki modeled on Wikipedia as a social as well as technological platform, where people could contribute and share knowledge, and together increase the total amount of accurate information available to users of the platform but also to the public. The CIAO project was a unique opportunity for the AOP proponents to see how the framework worked with a large team of scientists (40–50 at any one time), simultaneously collaborating. For this second question, we will reflect on the way in which values and motivations play out in the collaboration, the role of implicit modes of interaction, and how the AOP framework copes with these aspects.

## 3. Methods

To evaluate how effective is the AOP framework in supporting an *interdisciplinary* collaboration in the context of a viral disease and in working as the conceptual mediator of a c*rowdsourcing* model of collaboration, we used diverse methods: a survey disseminated among the CIAO participants, a workshop open to all interested CIAO contributors, a series of interviews with some participants and a self-reflection on the processes.

### 3.1. Survey

The survey was designed to explore the role played by the AOP framework, schematic and online Wiki in their experience as well as to identify the motivation and expectations of the participants joining the project. The survey was created as an online questionnaire using Google Forms web application, circulated among the CIAO participants from the 31th May 2021 until the 30th June 2021 (65 recipients at that date). The survey contained a total of 20 questions including Yes/No, multiple choice and free-text answers. The survey was divided in two sections: (i) experience in participating in the CIAO project (4 questions related to participant profile and 6 questions about reasons for joining the project), (ii) role of the AOP (framework, visuals and Wiki) in the CIAO collaboration (9 questions). The last question related to their potential interest in participating in the CIAO meta-level workshop.

### 3.2. Workshop

Workshop participants were recruited through an open call to all CIAO contributors across all working groups. The workshop was conducted online, using Zoom. A brief presentation of the results of the survey was followed by breakout groups. In each group, participants were asked to consider three aspects of the AOP framework: (i) the conceptual framework itself; (ii) the visualization of AOPs by diagram or schematic; and (iii) the AOP-Wiki as a tool for AOP development and knowledge management. Each breakout consisted of between 5 and 8 participants, and the participants were “re-shuffled” for each session (devoted to each one of the three aspects). The sessions were facilitated by a moderator, with a series of questions (Supplemental material) to scope how easy it had been to use the relevant aspect of the framework, whether there was agreement in their working group about the meaning or use of the aspect in question, if there was disagreement how it had been resolved, and if overall the aspect in question had added value to the collaboration.

### 3.3. Interviews of some participants

A series of semi-structured interviews focused on the experiences of interdisciplinarity in the project from 8 participants, who had either specifically talked about it in other contexts, or who were referenced by others in discussions (Questions in Supplemental material).

### 3.4. Reflection on our own processes

The coordination group consisting of representatives of all the working groups met regularly at roughly fortnightly intervals. The group discussed a wide range of issues relating to the whole project, including scientific, technical or social. In addition, the twice per year workshops, gathering all the working groups, were opportunities to reflect on the project, giving rise to many insights about how it is working as a collaboration. However, perhaps our best insights into the social aspects of the project and the hidden aspects of large crowdsourcing-based collaborations, occurred when there was a disruption in the collaboration. This led to the formulation of ground rules, which in itself was a process which entailed a high degree of self-reflection. This process is described in section 4.3.

## 4. Results

### 4.1. Survey

A total of 46 participants completed the online survey (out of 65 recipients, which was the number of participants in CIAO at the time of the survey). CIAO participants who completed the survey comprised experts from a variety of communities with most of them coming from academia (48%, *N* = 22), governance (17%, *N* = 8), public organization (13%, *N* = 6) or NGO (*N* = 4). Others respondents mentioned working in a hospital (*N* = 2), industry (*N* = 1), policy center (*N* = 1) or independently (*N* = 2).

**Figure 1:**
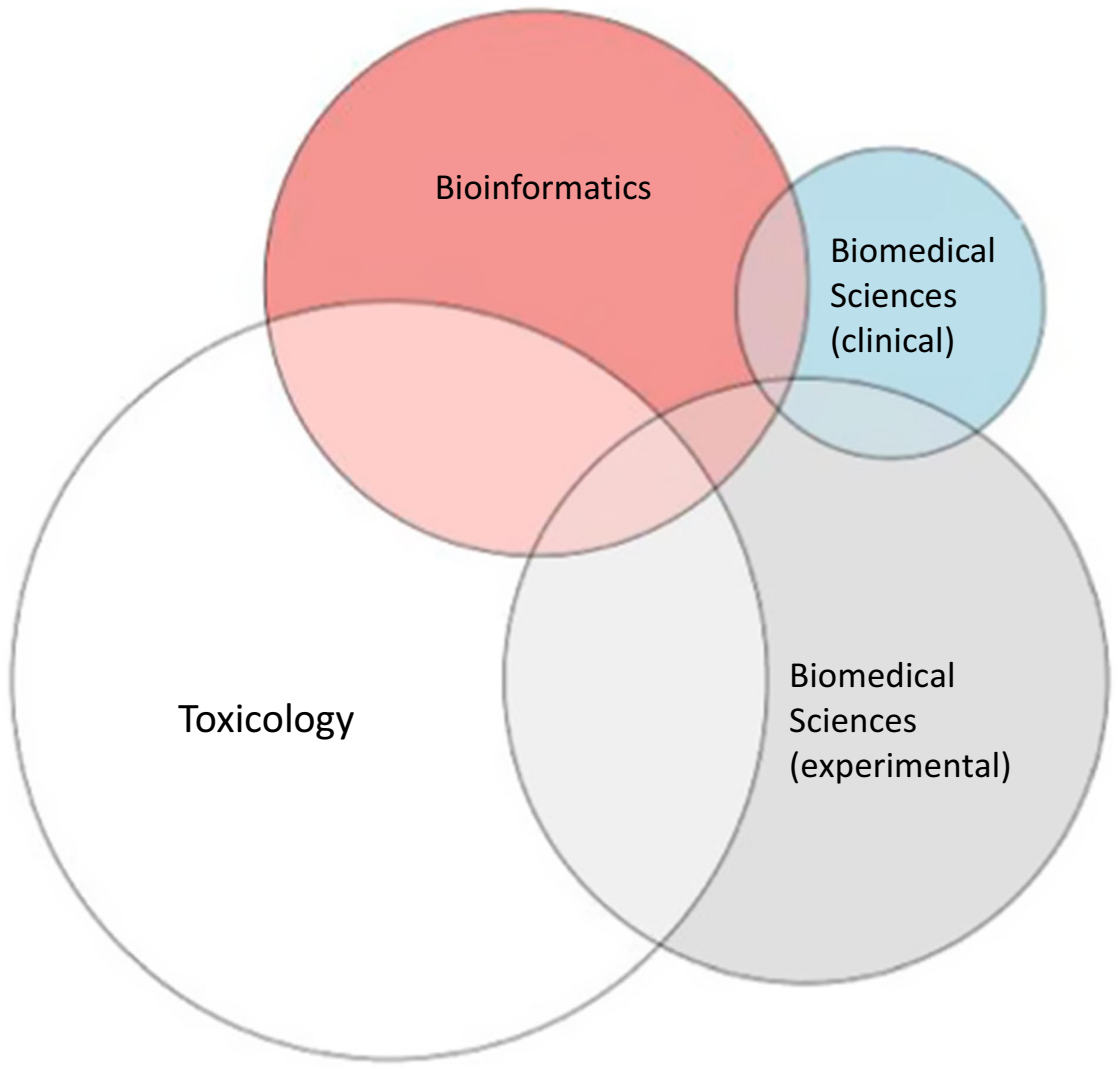
Overall span of professional expertise.

Toxicology represented 59% (*N* = 27) of CIAO expertise among the survey participants while biomedical sciences, experimental and clinical represented 35% (*N* = 16) and 11% (*N* = 5) respectively, followed by bioinformatics (28%, *N* = 13). Others mentioned systems biology, immunology, virology, genetics, comparative endocrinology, food safety (*N* = 1 for each), pharmacology (*N* = 2 for each) as their main expertise. No one declared expertise in epidemiology. More than half of the survey participants declared multiple expertise. The overall span of professional expertise is shown in Figure 1.

Among participants, 65% (*N* = 30) were women and 33% (*N* = 15) were men, which may be significant as research has shown that more women than men tend to collaborate in interdisciplinary projects (22, 23).

### 4.2. Workshop

The main points emerging from our discussions are described below.

#### 4.2.1. The AOP framework

**Figure 2:**
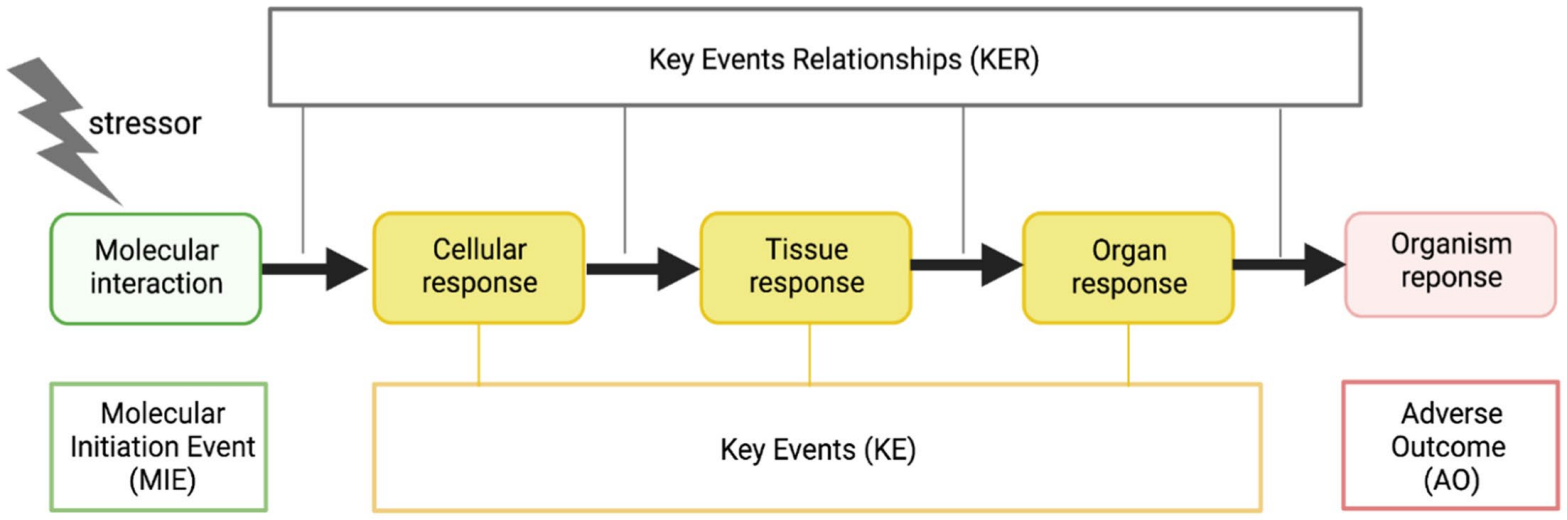
The AOP framework from Clerbaux et al. (4). Created with Biorender.com.

The core terminology of the AOP framework are the terms “Molecular Initiating Event” (MIE), “Key event” (KE), “Key event relationship” (KER), “Adverse Outcome” (AO), as an AOP traces a pathway from an MIE, through KE via KERs to an AO (Figure 2).

It was the middle terms (KE and KER) that participants experienced as the most difficult to apply in their own domains. While there are guidelines (24) and participants with previous expertise with the framework in each of the working groups, which are overall found to be very helpful in clarifying the concepts, participants still found it difficult to apply those concepts to concrete examples. Sometimes KEs are too generic, and sometimes too specific. They also found it difficult to distinguish between KEs and KERs. Based on this observation, a workshop was organized presenting the theory on how to build weight of evidence in KER and practical sessions where an expert in AOP development helped newcomers in AOP developing their COVID-19 KERs.

An AOP also moves across biological levels (cells, tissues, organs, whole organism), and participants found they had a lot of discussions on how to move between different levels. For example, there was a great deal of discussion as to whether an event that occurred at a “higher” level could count as an initiating event. While discussions could result in consensus around the biological plausibility of a KE or KER connection, there was less likely to be consensus on the weight of evidence for this connection. Notions such as temporality, causality and essentiality were much discussed. Other participants wanted a way to include variation between gene expression, protein levels and activity. Finally, many pointed to the need for an ontology to ensure common and harmonized terminology.

In addition, in the case of COVID-19, there are many modulating factors which may be biological (such as sex, age or a co-morbidity, like pre-existing heart failure) or extrinsic (such as diet or exposure to air pollution) that influence the outcomes of the disease. Many participants found this aspect was currently missing from the AOP framework, and advocated to integrate them. This was achieved in the CIAO project (4) resulting in a practical modus for including modulating factors to the AOP Wiki structure both on the AOP page^3^ and at the KER pages.^4^^,^^5^

Another intensely discussed aspect for the development of the COVID-19 relevant AOPs within CIAO, was the suitability of the principle that the AOPs are stressor agnostic. According to some proponents of the AOP framework, this principle in fact provides conditions for incorporation of different types of stressors in this toxicology-inspired framework because it calls for inclusion of evidence for perturbations (KEs) and linkages (KERs) without bias to the stressors that relate to the perturbations. This is complemented by the modularity principle, where KEs and KERs can be shared between AOPs leading to building networks. Despite the fact that elements from AOPs previously developed in the field of nanotoxicology (e.g., AOP173) were used as building block of some CIAO COVID-19 AOPs, there was robust discussion whether such stressor agnostic approach is suitable in the case of the COVID-19, a viral disease as depicting the biology of the virus is essential to understanding the disease.

Participants agreed that the AOP assisted with structuring information seeking:

But in the AOPs, I find that it's useful to sort of anchor that search because if you look for everything, you will find everything, right, you have millions of articles. But having this anchoring in […] what the experts think are key events and molecular initiating events […] that helps anchor the literature and design the structure, the literature search, so that it could [be] informative of the pathway.

In terms of achieving consensus, this was clearly easier for some points rather than others. In addition, sometimes sufficient consensus for continuing the discussion was obtained, and this was sometimes influenced by the presentation of a visual graphic by one of the members of a working group. This tended to give stability to the discussion or even to become the dominant interpretation (more on this in section 4.2 below).

The working groups meet regularly, and discussions occur over weeks and months. As one participant put it, “it’s really an evolving process,” that involves putting into practice as much as discussing:

We found that the practice answering evidence teaches us further about the concept”—“it's the application of a concept to a concrete example that is really helping out in understanding exactly, if something is a key event versus a relationship or an adverse outcome or a molecular initiating event”

Even though there was not consensus on all points, participants did not find this a block to discussion. They pointed out that the discussion generated by not immediately all agreeing helped to open new ways of thinking:

So I think having those discussions and breaking it down like that, and learning from other people in different areas, I think that was very helpful. So at times, there's perhaps, you know, we didn't all agree because you know […] it makes you think, in a different way.

Thinking in a different way can be masked by the different terms and vocabularies used by people from different backgrounds:

And there is […] a lot of ontologies and vocabulary issues [...] which I find very, very interesting, because sometimes we talk about the same thing, but it's called differently or the other way around.

Discovering those differences through a common framework that all are trying to map their knowledge onto, can also assist interdisciplinary collaboration.

Sometimes the lack of consensus was useful, because it helped to identify where there are gaps in knowledge as well as different perspectives, which is crucial for productive interdisciplinary collaboration. Participants generally agreed that using the framework helped them to assemble knowledge across the wide range of different specialisms in working groups:

I definitely think it helped in collaboration and it also helped us agree and communicate on a similar level in terms of, especially among the different groups being able to discuss a certain event as either a key event or an outcome, and then seeing how those overlap between the different systems.

Others mentioned that this was a two way process in that the framework helped them to collaborate—because it provides a system of terms and concepts that acts as a focus for discussion -, but also having collaboration made the AOP they were jointly working on more reliable. At some points, suggestions were made as to how the framework could be developed, for example to have a generic title for a KE, with sub-titles, to deal with the more specific aspects. This two-way relationship whereby the framework structures discussion, but at the same time is refined and modified by that discussion, is typical of what we would expect from a framework that mediates a process.

The points on which there is most discussion, or remaining open questions, are similar to those that are raised by the AOP framework in its “home” domain, which shows that the conceptual challenges of the framework are not domain specific. The issues that arise most sharply when the framework is applied for real world decision making in toxicology, specifically those around including information about stressors, quantitative aspects and groups of similar chemicals—clearly emerge in a disease domain even more urgently where biological variants emerge in a rapid and much less predictable manner. The issue of modulating factors is extremely significant in a disease domain, and particularly in a disease such as COVID-19. A conceptual challenge coming from a representative of biomedical practitioners among the CIAO participants was to include beneficial outcomes as well as adverse outcomes.

#### 4.2.2. The AOP visuals

The standard AOP diagram is shown in Figure 1. The diagram is frequently a focal point of discussion among collaborators. The question that invariably arises is “*how simple or complex it should be*,” with differing answers depending on the domain, discipline and purpose. This issue was prominent in CIAO discussions. For example, one participant said:

It's really difficult because it never, there's never just one pathway leading to cancer, you have multiple pathways, and I'm just struggling with the fact […] if I build one AOP from one of those paths, but I don't include information that there are other paths leading to or simultaneously synergistically working together towards that adverse outcome, then there's, that's a problem. That's a bit of an issue. And I don't know how to [...] solve that. And I think we have a similar situation in COVID-19.

The linearity of the diagram is also frequently mentioned as a limitation. Several participants talked of the need to consider feedback loops and networks. Making the diagrams more interactive is also a request frequently made by users, and CIAO participants were no different, asking for interactivity that would allow different levels of complexity and information depending on needs, and interactivity that would allow manipulation and different “what if” scenarios. These are comments on the AOP visualization that are frequently made in its home domain of toxicology too, and ongoing development aims to address these issues.

Many participants pointed out the relative visual imbalance between KEs and KERs, and wanted more prominence given to the KERs, where a lot of crucial information comes in. One suggestion was to replace the arrow with an evidence table, or to make it more interactive, clicking on the line would open an evidence table. Other suggestions were to indicate the strength of evidence for a relationship visually as well, for example through the thickness of the line. The question of what counts as evidence that would make the line thicker was also discussed, since it should not only be the number of papers about the relationship. Other participants would not want the line and arrow completely replaced, as it conveys causality. However at the same time, each of these notions were also discussed as it is by no means easy to convey them visually, and at times difficulties with those concepts underlies the difficulties of visual representation. In diseases, it is especially difficult to distinguish sequences of events, because many processes in pathophysiology can occur in parallel and do not fit into a linear sequence. The conceptual limitation of the AOP framework in relation to MIE also comes out very clearly in the diagram, with some participants asking “*what about pre-MIE*?” While there is questioning of the molecular approach of the AOP framework even in toxicology, this is an issue that comes out even more clearly for the range of disciplines brought in by the CIAO project, many of which want to consider the pathways of a disease such as COVID-19 in a more contextualized way. For example, this fits in with the concerns about how to bring modulating factors into the AOP, and also into the diagram; and it also fits with concerns to consider the disease from social or economic perspectives, as well as biologically.

Most participants agreed that the diagrammatic mapping out of the pathways is crucial to the process of developing AOPs and represent them in an intuitive and accessible way. It helps to structure discussion and work processes and to show up where there are gaps. The diagrams were also found to be very useful for the collaboration. Similarly as for the AOP as a conceptual framework, the diagram was described as anchoring the discussion. This participant described the diagram as particularly helpful in bringing together different perspectives:

When you start visualizing these things, you start to see how other people are viewing things. And I think certainly from a collaborative point of view [...] that’s very helpful [...]. And it’s also I think, it’s interesting to see different people’s backgrounds, because everybody, all of us approach things [...] in different ways, because of our backgrounds…”

Others agreed that the diagrams are a useful way to enable others to understand what you are talking about. In the quote below, the participant mentions several different aspects of the diagram: it allows for separate working from different perspectives, comparison between perspectives, communication, and giving a summarized overview of a lot of dispersed information.

People can work simultaneously. And they each have their own starting point or building blocks in between. And then if you compare that later on, you get a lot more information. It’s a nice tool to communicate also, because it simplifies, it’s much easier to see different points of view in an AOP manner than then again, going through hundreds of pieces of papers.

However, as noted above, it is also possible for a diagram that is offered by one contributor to a working group both to stabilize discussion, and also to become dominant, simply because there are not others. This can also potentially create its own blindspots. The importance of also using other diagrams was noted, not only other diagrams of the pathway(s) in question, but other genres of diagrams entirely, such as multiscale depictions, or bullseye depictions.

#### 4.2.3. The AOP-Wiki

The AOP-Wiki is a knowledge sharing platform publicly accessible, which allows people to access and to contribute knowledge.^6^ It is the indispensable complement to the AOP framework. The framework provides the principles for organizing knowledge and the Wiki provides the means for sharing in an open access manner the knowledge. The AOPs in the AOP-Wiki provide a synthetic overview of the accumulated knowledge about the elements of each pathway and where there is potential for connections between pathways. As our participants said, it makes the knowledge about pathways more accessible, both in the sense that all is in one place rather than distributed and also easier to understand as it is organized.

CIAO provided a practical large-scale multidisciplinary “stress test” of the main development and communication tool of the AOP framework. Several of the participants in the workshop were using the AOP-Wiki to upload their own AOPs as they are being developed, as this is the major aim of the CIAO project. As such they were able to talk about the Wiki as developers (rather than users). The feedback from participants on the Wiki reiterated points that have been made in the AOP home domain, notably that uploading AOPs can be burdensome and difficult if one is not already well versed in the AOP conceptual framework, in particular because there are many text boxes in the template. Participants said that this makes the uploading of AOPs time consuming, while at the same time making it difficult to achieve standardization. One participant said that possibly there are too many degrees of freedom in the text boxes. The most frequent call is for more standardization with respect to vocabulary and terminology, and for a clear ontology. Participants also remarked on the need for a way of presenting evidence and levels of certainty geared toward different audiences, such as regulators, or other stakeholders who may be in a position to use the knowledge on the Wiki to inform their decisions, or specific decisions.

In line with previous comments about KEs and KERs, the advice from our participants is not to proliferate KEs, but to concentrate on KERs, which is where evidence is critical. This is also connected to the issues with the diagrams too easily lending themselves to the dominance of KEs over KERs. Once again we heard the need for evidence tables, this time to replace text boxes, making the AOP less burdensome to upload, and at the same time, making the evidence for a KER clearer.

Just as modulating factors are difficult to represent in the diagrams, they were also difficult to include in the Wiki. There was no space for them in the AOP template, and they therefore go into free text, which makes them more peripheral to the AOP framework, limiting its usefulness as a framework for sharing knowledge about diseases as this participant pointed out:

“... my personal view is that now that we are expanding the AOP to include those two potential … pathophysiology overlapping with [....] toxicology, I think [...] the role played by the modulating factor is ever more important sometimes to determine whether the system will shift towards the AO or not. So capturing that information may be probably very useful”

This was seen as a serious problem by another participant, who said:

“This idea of modulating factors is fundamentally a serious problem because there is a continuum between what we call an event and a modulating factor. And things that we think are events, sometimes, especially if you think about COVID [..] things that are modulating factor can mean the difference between [...] life and death”

Extending the AOP framework and its supporting tool, the AOP-Wiki, to the challenge of sharing data and knowledge about disease pathways identified the need for a re-think about some of its basic assumptions, including what counts as key events. Most importantly, the Wiki needed to reflect the role of modulating factors. Tables dedicated to modulating factors have since been included in the Wiki at the AOP and KER level pages, thanks to the CIAO recommendations. These tables were completed by the CIAO members of the modulating factors working group to test and eventually adjust them via the “learning by doing” principles in a successful bidirectional collaboration between Wiki users (CIAO) and those responsible for the Wiki. This is what we would expect from a system that plays a mediating role, as it shapes the domain it acts in, but is also shaped by it.

Table 1 summarizes the challenges and advantages of using the AOP framework, diagram and AOP-Wiki to drive interdisciplinary collaboration as identified by the CIAO participants.

**Table 1:** Advantages and challenges of the AOP framework, visuals and AOP-Wiki in driving interdisciplinary collaboration.

Framework	
Advantages	Stressor agnostic approach leading to knowledge sharing between disciplines, Cover the different biological levels associated to different disciplines, Foster focused and structured discussions.
Challenges	Describe virus biology while staying compliant with the stressor agnostic approach, Move between the different biological levels, Need for ontology to ensure common and harmonized terminology.
Visuals	
Advantages	Capture essential steps from dispersed information sources, Show and make explicit different perspectives and assumptions, Represent an intuitive and accessible way to develop an AOP, Help to structure discussions and mediate consensus.
Challenges	Linearity of the AOP diagram for complex biological processes, Balance between comprehensive detail and informative simplicity, Consideration of “pre-MIE,” i.e., exposure, Visual imbalance between KEs compared to the KERs, Difficult to represent modulating factors in the diagram.
AOP-Wiki	
Advantages	Living documents that can be updated when new information become available, Flexibility to accommodate modifications when needed, Shared online publicly accessible platform for collaboration, Show up gaps across the different biological levels.
Challenges	AOP development within the AOP Wiki is a resource intensive process, Difficult to achieve standardization of free text field entries, Work needed to devise suitable and effective evidence tables, Duplication of KEs: needs for harmonization of concepts and ontology.

### 4.3. Interviews of CIAO participants

Frequently the interdisciplinarity of projects is in name only, and it only rarely occurs that there is a collaboration that leads to the mutual change, the hallmark of genuine interdisciplinarity. In interviews, participants were asked about their experience of interdisciplinarity. Their responses showed that a major motivator for them to be involved was the level of interdisciplinarity the project afforded. As one participant put it: “*how to use the framework makes you constantly think and reflect, also on your own ideas and perhaps also on your own biases*.” There were many different forms of interdisciplinarity in the project, but the following types stand out.

The collaboration between a nanotoxicologist well versed in AOPs and a virologist with no prior knowledge of the AOPs resulted in discovering commonalities and differences between toxicology and virology, leading to a new understanding of how dose response and stressors operate in a similar manner in toxicity and in virology once you focus on biological pathways.

The collaboration between clinicians and biomedical researchers brought about what might be the most important change in the AOP approach: the new prominence given to modulating factors (vitamin D deficiency, diet, sex, age). The complexity of the interconnections between modulating factors became visible only through the application of the AOP framework by a number of scientists with different expertise, able to illuminate what were the interactions among modulating factors and the biology.

Bringing together lab based and computational methods resulted in making progress in moving from the Wiki based knowledge sharing platform, including diagrammatic visualizations of pathways, to graphs that can be used for computational modeling, through iterative loops between experimentalists curating the knowledge sharing platform, and a modeler.

Finally, the collaboration with those who brought expertise in public health and specialists in social aspects of medicine brought attention to gender and socio-economic status. There were also multiple conversations about the molecular focus of the AOPs, and one working group made some progress on considering a multiscale approach that would better contextualize AOPs in a broader framework.

### 4.4. Reflections on knowledge-sharing collaborations

Proponents of the AOP framework and Wiki for sharing knowledge have used the model of the crowdsourcing approach since their inception. There is no direct reward for contributing to the Wiki. A strong motivation is expected to be that of participating in a joint effort to build up AOPs, from which contributors would gain in many different ways (19). The CIAO project similarly was born of a motivation to take this cooperative knowledge sharing motivation to help tackle one of the most significant global health challenges. Great emphasis was placed on the potential of bringing together a community of people from many different backgrounds and disciplines, in order to tackle a disease as complex as COVID-19. The emphasis soon shifted from crowdsourcing to community, as participants were actively involved in regular meetings, and got to know each other. The approach of the project has always been very inclusive and open. From the outset, it was known that participating in any of the working groups was a task that members took on either within their normal working duties, or over and beyond these. Altruism, professionalism, and helpfulness are dominant values of the community, alongside of course, intellectual curiosity, new insights, etc. Typically, participants were members of one or more working groups, who collaborated on a more or less informal footing.

#### 4.4.1. Emergence of ground rules

The four workshop reports published for externals or circulated internally (14, 25) were conceived as ways of sharing the progress of the project within the CIAO community and with a wider audience following the workshops. At first, the authorship was restricted to members of the coordination group. Then in the interests of greater inclusivity, it was decided to extend it to all the members of CIAO who had contributed to some aspect of the results presented at the workshop. At this point, all co-authors had to formally fill in their affiliations, and it became clear that one of the contributors was employed by the tobacco industry. This gave rise to disquiet among some participants, who for different reasons, would not or could not appear in a co-authorship list that included a tobacco company. There were many discussions and the workshop report was held up for some time.

This disruption was useful in bringing to the surface some of the assumptions made in setting up the crowdsourcing collaboration, and the different and sometimes conflicting expectations of the community. There were very different attitudes to the connection between contributors and the organizations they are affiliated with. Some saw the exclusion of a person due to affiliation as a form of discrimination; others were constrained by their own organization from co-authoring with someone from a tobacco company; and yet others felt strongly that especially because COVID-19 is a disease that is exacerbated by tobacco, there should be a strong stance against the tobacco industry, extending to possibly making this a theme of enquiry in the project. The differences of opinions were clearly embedded within the wider value systems of contributors to the project, including aspects such as the social responsibilities of scientists; the extent of intellectual freedom of scientists and where or how this freedom is expressed; the potential for science to be biased by interests. As the disagreement focused on the issue of co-authorship and membership of the community, we realized that we needed first, a means of ensuring that there is an explicit and shared understanding of some core ground rules for the project, and second, a procedure for dealing with disagreement. In order to tackle this, we drew up a document setting out a proposal for ground rules. The process of setting up the ground rules was itself an opportunity for self-reflection, as it revealed our own expectations of the AOP project, of crowdsourcing, and of the scientific community. The ground rules document went through different versions, as drafts were opened to feedback from the coordination group in the first rounds, and all the other working groups in subsequent rounds, before being accepted.^7^

Several aspects of the ground rules deserve comments. However we will focus on just a few. The ground rules are a compromise between the different perspectives of its members, and try to balance an open membership approach (necessary to achieve real interdisciplinarity) with the right of members to decide with whom they wish to collaborate, especially when that affects co-authorship.

1. The potential membership of the crowd was construed very widely, with a non-discrimination clause, followed by a clause explicitly saying that professional affiliation with any particular organization would not be an obstacle to membership. This is in line with the values of openness of the project, effectively declining to exclude people because of their affiliation to any particular industry.
2. Along with this went an obligation to disclose potential conflicts of interest on becoming a member. This is in line with the value of transparency that all members subscribe to. It would also address the issue that people should know about potential conflicts in advance of a collaboration, and not only once it has progressed to publication stage.
3. Contributing to the crowd—via participation in working groups and contributing information in the Wiki (for example)—is distinct from co-authoring. Co-authoring teams are essentially sub-groups of working groups, and the prior disclosure of conflicts of interest mean that the teams are formed around people’s co-authoring constraints.
4. All members can contribute to the AOP-Wiki. The combination of the declaration of conflicts of interest, together with the collaborative process of checks and balances among members of a working group (or sub-group working on a specific AOP), together with the peer review processes already included in the AOP-Wiki, were felt to be sufficient to avoid bias or misinformation.

Recalling that one of our questions is about the capacity of the AOP framework to support collaborations beyond its initial remit, writing a version of the ground rules acceptable to all also saw a continuation of the discussion about the remit of the project, particularly in the background section setting out the goals and scope. The extent of the interdisciplinary breadth of the project also came up for renewed discussion: the AOP framework maps biological pathways, to what extent can it or should it be opened up to broader bio-social perspectives? Another question that came up was how engaged the project should be with the aspects of the disease that are related to corporate or political bodies. For example, to what extent should it engage in any way with particular industries or agencies, rather than, for example, tobacco or air pollution in general? The agreed text now refers to an extension of the AOP framework from its origins in toxicology to the biomedical domain. This includes consideration of modulating factors, but also broader social, economic, and environmental aspects that influence the course of the disease to be considered in a more holistic framework (26). Maintaining the biological focus of the project while broadening the scope to these other determinants of the disease was a compromise between different views on how action on the disease should be taken. The list of potential stakeholders in the project who may benefit from the knowledge shared by the project is broad, and includes health care providers, public health specialists, health policy makers and pharmaceutical companies. In this way the project indicates neutrality about the different approaches that can be taken to the disease. It is by no means the only way it could be achieved, and it does not offer itself as a closure of the debate. For example, the working group that deals with the broader contextualization of the disease, and of the framework itself (the so-called multiscale or “rogue” group), continue to be part of the project, and there continue to be discussions about broader context for the disease. However, this compromise which offered to maintain the openness of the project to as wide a crowd as possible reached a majority approval by those who were members of the crowd in September 2021. Everyone who wished to remain a member of the crowd was asked to sign that they agreed to the ground rules, as well as to fill in the conflict of interest declarations. Seventy eight had signed the rules by January 2022. Only the one who made the initial objection did not agree to sign, as he felt that the ground rules did not address his concerns.

The ground rules also contain a section detailing a procedure to follow in the case of dispute. This acknowledges the potential for dispute, which had previously not been addressed, simply because it had not occurred. The initial framing of the project, as an opportunity to pool together to share knowledge about COVID-19 using the AOP framework, assumed a common mind-set and value system. This was the implicit horizon of expectations for the project, and indeed the project has been initiated, established and carried forward on a groundswell of generous and altruistic support. That a dispute emerged does not negate this, but rather shows up how different are the value systems that jostle together in the crowd of a crowdsourcing project. Having a way of acknowledging and dealing with these aspects is crucial to the management of any crowdsourcing project. Ideally ground rules should be associated with the beginning of the project.

The discussions over the remit of the project were demanding for all those who participated, intellectually and emotionally. This is, after all, a project, which most people are contributing to in their spare time, without any immediate benefit in sight. The discussion itself was an exercise in self-reflection for everyone concerned, showing up scientific, pragmatic and ethical values of those who participated directly in it. The solution found in setting out ground rules is an attempt to ensure transparency from the outset, and an acknowledgement of the complexity hidden under a common desire to make a useful contribution to addressing a serious disease.

## 5. Discussion

The background hypothesis that motivated the idea for the CIAO project was that AOPs could have great value for biomedical research, assisting with a transition to a more evidence-based understanding of the underlying mechanisms of a disease. Motivated by the prospect of doing something useful at a time when the COVID-19 pandemic had brought about a global crisis, the CIAO project also became a living experiment in testing how effective the AOP framework is in supporting an *interdisciplinary* collaboration on the topic of a viral disease. This research question contains two interlinked aspects: interdisciplinarity and adaptation of a toxicity-based framework to a viral disease.

The framework provided a common reference point to structure discussion and achieve interdisciplinary collaboration as it covers the different biological levels usually associated with different disciplines (e.g., molecular and cellular level for lab researcher, tissue or organ level for clinicians). The stressor agnostic approach allows bridging the knowledge of pathway and adversity between disciplines. However, how to move between the different biological levels and how to describe the viral stressor that undergoes its own dynamic biological transformation, while staying stressor agnostic, remain challenging. CIAO participants also pointed the need for an ontology to ensure common and harmonized terminology to help refinements of concepts in the future. Of great interest, modulating factors came to a new prominence in tracing mechanistic pathways of disease. It will be interesting to see how the knowledge gained about modulating factors could be re-used and useful for toxicity pathways. The diagrams commonly used in the AOP framework assist with making explicit what are the different perspectives brought to knowledge about the pathways and represent an intuitive way to develop an AOP collaboratively while helping to achieve consensus. At the same time their use for this collaboration showed up which aspects are still challenging such as balancing between comprehensive detailed complexity and informative simplicity but as well how to integrate exposure and how to represent modulating factors in the diagram.

The use of the AOP-Wiki by a big crowd around a common but complex project showed the great potential about its usability for those not already in the world of AOPs, but also highlighted some challenges for all. AOPs are living documents that can be updated when new information become available and are shared via an online publicly accessible platform fostering collaboration. However, uploading AOPs into the AOP-Wiki is time consuming. CIAO participants also pointed the needs for more machine-readable evidence table to replace free text and for standardization and ontology harmonization. Making space for modulating factors was potentially the most disruptive adaptation for the AOP-Wiki. In addition, the experience gained with the use of the AOP-Wiki in CIAO, continues to inform future development of this collaborative platform and potentially the framework itself. Incorporating features that enable tracking provenance of content in the AOP-Wiki and “citability” of AOPs developed within the platform also relate to social aspects of crowdsourcing within the Wiki. Another potentially disruptive adaptation for the AOP-Wiki and/or the framework could precipitate from the particular challenge with including viral and other biological stressors (Clerbaux et al. in preparation). The collaborating platforms (the enabling technologies) have significant role in driving both, social and technical aspects of crowdsourcing, including in biomedical science (27, 28).

Most importantly, the project has supported genuine interdisciplinarity where there has been an exchange of knowledge that results in novel insights, and novel research directions. Starting with the motivation to make a contribution, the excitement and stimulation of being in an interdisciplinary collaboration is what kept participants on board.

Regarding how well the AOP framework supports a *crowdsourcing* model of collaboration, this is a question that goes beyond the framework alone, and encompasses all the organizational effort that goes into establishing and maintaining a large-scale collaboration across disciplines and countries. The success of any crowdsourcing initiative is likely related the ease of use, potential to incentivize crowd contribution, the ability to allow for efficient processing of the data contributed by the crowd and its visualization to communicate within and beyond the crowd. Interdisciplinary crowd-based projects like CIAO may face additional challenges as they aim to capture different types of data, lines of evidence and approaches to their analysis. The CIAO project shows that this is successful when there is a strong central organizational impetus and ongoing support, supplied in this instance by the JRC Systems Toxicology Unit. It also requires attention to be paid to the different values, interests and purposes of the participants. The point when a formal agreement is required will not be the same for different projects. When embarking on a large-scale collaboration of this sort, clarity about the terms of the collaboration (“ground rules”) is something that is best brought about as early as possible, and certainly not assumed as implicit.

## 6. Conclusion

The experience of the CIAO project has many lessons for interdisciplinary collaboration. It shows the essential roles of motivation, strategic leadership, and focused project management in establishing and sustaining interdisciplinary collaboration. In addition, it shows the importance of building in ways to bring to the surface implicit assumptions, about science, and about the social organization of the collaboration. It exemplifies how the most common barrier to interdisciplinarity—the absence of a shared language—can be addressed through the use of a conceptual framework such as the AOP framework. There are promising prospects for deploying the CIAO approach and related processes to other areas of science where the AOPs can foster true interdisciplinary and systematic organization of existing knowledge. Of immediate interest are long COVID or post-acute COVID-19 syndrome, currently estimated to affect 65 million people worldwide. To understand the mechanisms of long COVID and to identify which factors render some individuals more susceptible, a similar degree of scientific collaboration across disciplines needs to be maintained. An AOP-aligned interdisciplinary collaborative crowdsourced approach could be instrumental in these areas. Of even wider interest would be the use of this approach way beyond health fields.

## Data availability statement

The original contributions presented in the study are included in the article/supplementary material, further inquiries can be directed to the corresponding author.

## Ethics statement

Ethical review and approval was not required for the study on human participants in accordance with the local legislation and institutional requirements. Written informed consent from the participants was not required to participate in this study in accordance with the national legislation and the institutional requirements.

## Author contributions

AC, CW, and L-AC prepared, disseminated, analyzed the survey, organized, and analyzed the workshop. AC performed the interviews and conceptualized the manuscript. CW developed the ground rules with the help of AC and of the CIAO coordination group. All authors contributed to the writing of the manuscript, read, and agreed to this version of the manuscript.

## Funding

This work was supported by the European Commission Expert (Contract No. CT-EX2017d316740–101).

## Conflict of interest

The authors declare that the research was conducted in the absence of any commercial or financial relationships that could be construed as a potential conflict of interest.

## Publisher’s note

All claims expressed in this article are solely those of the authors and do not necessarily represent those of their affiliated organizations, or those of the publisher, the editors and the reviewers. Any product that may be evaluated in this article, or claim that may be made by its manufacturer, is not guaranteed or endorsed by the publisher.

## Footnotes

^1^ https://www.ciao-covid.net/aops

^2^ https://aopwiki.org/

^3^ https://aopwiki.org/aops/468

^4^ https://aopwiki.org/relationships/1703

^5^ https://aopwiki.org/relationships/2354

^6^ https://aopwiki.org/

^7^ https://www.ciao-covid.net/groundrules

## References

[1] Nymark P Sachana M Leite SB Sund J Krebs CE Sullivan K. Systematic organization of COVID-19 data supported by the adverse outcome pathway framework. Front Public Health. (2021) 9:638605:1–12. doi: 10.20944/preprints202101.0573.v1 34095051 PMC8170012

[2] Ankley GT Bennett RS Erickson RJ Hoff DJ Hornung MW Johnson RD. Adverse outcome pathways: a conceptual framework to support ecotoxicology research and risk assessment. Environ Toxicol Chem. (2010) 29:730–41. doi: 10.1002/etc.34, PMID: 20821501

[3] Villeneuve DL Crump D Garcia-Reyero N Hecker M Hutchinson TH LaLone CA. Adverse outcome pathway (AOP) development I: strategies and principles. Toxicol Sci. (2014) 142:312–20. doi: 10.1093/toxsci/kfu199, PMID: 25466378 PMC4318923

[4] Clerbaux LA Albertini MC Amigó N Beronius A Bezemer GFG Coecke S. Factors modulating COVID-19: a mechanistic understanding based on the adverse outcome pathway framework. J Clin Med. (2022) 11:4464. doi: 10.3390/jcm11154464, PMID: 35956081 PMC9369763

[5] Clerbaux LA Amigó N Amorim MJ Bal-Price A Leite SB Beronius A. COVID-19 through adverse outcome pathways: building networks to better understand the disease - 3rd CIAO AOP design workshop. ALTEX. (2022) 39:322–35. doi: 10.14573/altex.2112161, PMID: 35032963 PMC10069302

[6] Clerbaux LA Fillipovska J Muñoz A Petrillo M Coecke S Amorim MJ. Mechanisms leading to gut dysbiosis in COVID-19: current evidence and uncertainties based on adverse outcome pathways. J Clin Med. (2022) 11:5400. doi: 10.3390/jcm11185400 36143044 PMC9505288

[7] Hogberg HT Lam A Ohayon E Shahbaz MA Clerbaux L-A Bal-Price A. The adverse outcome pathway framework applied to neurological symptoms of COVID-19. Cells. (2022) 11:3411. doi: 10.3390/cells11213411, PMID: 36359807 PMC9658029

[8] Pistollato F Petrillo M Clerbaux L-A Leoni G Ponti J Bogni A. Effects of spike protein and toxin-like peptides found in COVID-19 patients on human 3D neuronal/glial model undergoing differentiation: possible implications for SARS-CoV-2 impact on brain development. Reprod Toxicol. (2022) 111:34–48. doi: 10.1016/j.reprotox.2022.04.011, PMID: 35525527 PMC9068247

[9] Shahbaz MA De Bernardi F Alatalo A Sachana M Clerbaux L-A Muñoz A. Mechanistic understanding of the olfactory neuroepithelium involvement leading to short-term anosmia in COVID-19 using the adverse outcome pathway framework. Cells. (2022) 11:3027. doi: 10.3390/cells11193027, PMID: 36230989 PMC9563945

[10] Vinken M. COVID-19 and the liver: an adverse outcome pathway perspective. Toxicology. (2021) 455:152765. doi: 10.1016/j.tox.2021.152765 33771662 PMC7986318

[11] Burtt JJ Leblanc J Randhawa K Ivanova A Rudd MA Wilkins R. Radiation adverse outcome pathways (AOPs) are on the horizon: advancing radiation protection through an international horizon-style exercise. Int J Radiat Biol. (2022) 98:1763–76. doi: 10.1080/09553002.2022.2121439, PMID: 36067511

[12] Halappanavar S Van Den Brule S Nymark P Gaté L Seidel C Valentino S. Adverse outcome pathways as a tool for the design of testing strategies to support the safety assessment of emerging advanced materials at the nanoscale. Part Fibre Toxicol. (2020) 17:1–24. doi: 10.1186/s12989-020-00344-4 32450889 PMC7249325

[13] Sachana M Leinala E. Approaching chemical safety assessment through application of integrated approaches to testing and assessment: combining mechanistic information derived from adverse outcome pathways and alternative methods. Appl In Vitro Toxicol. (2017) 3:227–33. doi: 10.1089/aivt.2017.0013

[14] Wittwehr C Amorim MJ Clerbaux L-A Krebs C Landesmann B Macmillan DS. Understanding COVID-19 through adverse outcome pathways – 2nd CIAO AOP design workshop. ALTEX. (2021) 38:351–7. doi: 10.14573/altex.2102221, PMID: 33677612

[15] Hosseini M Shahri A Phalp K Taylor J Ali R. Crowdsourcing. Comput Sci Rev. (2015) 17:43–69. doi: 10.1016/j.cosrev.2015.05.001

[16] Estellés-Arolas E González-Ladrón-de-Guevara F. Towards an integrated crowdsourcing definition. J Inf Sci. (2012) 38:189–200. doi: 10.1177/0165551512437638, PMID: 33036834

[17] Brabham DC Ribisl KM Kirchner TR Bernhardt JM. Crowdsourcing applications for public health. Am J Prev Med. (2014) 46:179–87. doi: 10.1016/j.amepre.2013.10.016, PMID: 24439353

[18] Uhlmann EL Ebersole CR Chartier CR Errington TM Kidwell MC Lai CK. Scientific utopia III: crowdsourcing science. Perspect Psychol Sci. (2019) 14:711–33. doi: 10.1177/1745691619850561 31260639

[19] Carusi A Davies MR De Grandis G Escher BI Hodges G Leung KMY. Harvesting the promise of AOPs: an assessment and recommendations. Sci Total Environ. (2018) 628–629:1542–56. doi: 10.1016/j.scitotenv.2018.02.015 PMC5888775 30045572

[20] Morgan MS Morrison M. Models as mediators: perspectives on natural and social science Cambridge University Press (1999).

[21] Carusi A. *In silico* medicine: social, technological and symbolic mediation. Hum J Philos Stud. Cambridge University Press (2016) 30:67–86.

[22] Rhoten D Pfirman S. Women in interdisciplinary science: exploring preferences and consequences. Res Policy. (2007) 36:56–75. doi: 10.1016/j.respol.2006.08.001, PMID: 34417188

[23] Van Rijnsoever FJ Hessels LK. Factors associated with disciplinary and interdisciplinary research collaboration. Res Policy. (2011) 40:463–72. doi: 10.1016/j.respol.2010.11.001, PMID: 36726618

[24] OECD. User’s handbook supplement to the guidance document for developing and assessing AOPs. OECD Environment, Health and Safety Publications (2018).

[25] Clerbaux LA Mayasich SA Muñoz A Soares H Petrillo M Albertini MC. Gut as an alternative entry route for SARS-CoV-2: current evidence and uncertainties of productive enteric infection in COVID-19. J Clin Med. (2022) 11:5691. doi: 10.3390/jcm11195691 36233559 PMC9573230

[26] Vineis P Barouki R. The exposome as the science of social-to-biological transitions. Environ Int. (2022) 165:107312. doi: 10.1016/j.envint.2022.107312 35635963

[27] Khare R Good BM Leaman R Su AI Zhiyong L. Crowdsourcing in biomedicine: challenges and opportunities. Brief Bioinform. (2016) 17:23–32. doi: 10.1093/bib/bbv021, PMID: 25888696 PMC4719068

[28] Lenart R Wojciech G Łukasz C. Understanding crowdsourcing in science. Berlin Heidelberg: Springer (2022).

