# Supplementary material for: CIAO: a living experiment in interdisciplinary large-scale collaboration facilitated by the Adverse Outcome Pathway framework
Source: Front Public Health. 2023 Aug 10;11:1212544. doi: 10.3389/fpubh.2023.1212544 (PMC10449328; doi:10.3389/fpubh.2023.1212544)
Supplement: Supplementary file 1 [file Data_Sheet_1.PDF]

Supplementary Material

TITLE: Life on the Wall: The Diversity and Activity of Microbes on 13th AD. Lan Na Mural Painting

Chakriya Sansupa [1,¶], Nattaphon Suphaphimol [1,¶], Paradha Nonthijun [1], Teewararat Ronsuek [1], Saranphong Yimklan [2], Natthawat Semakul [2], Thapakorn Khrueraya [3], Nakarin Suwannarach[1, 4], Witoon Purahong [5,*] and Terd Disayathanoowat [1,4*]

¶ These authors share first authorship

*** Correspondence:**

Witoon Purahong

witoon.purahong@ufz.de (W.P.)

Terd Disayathanoowat

Terd.dis@cmu.ac.th (T.D.)

Supplementary Figures and Tables

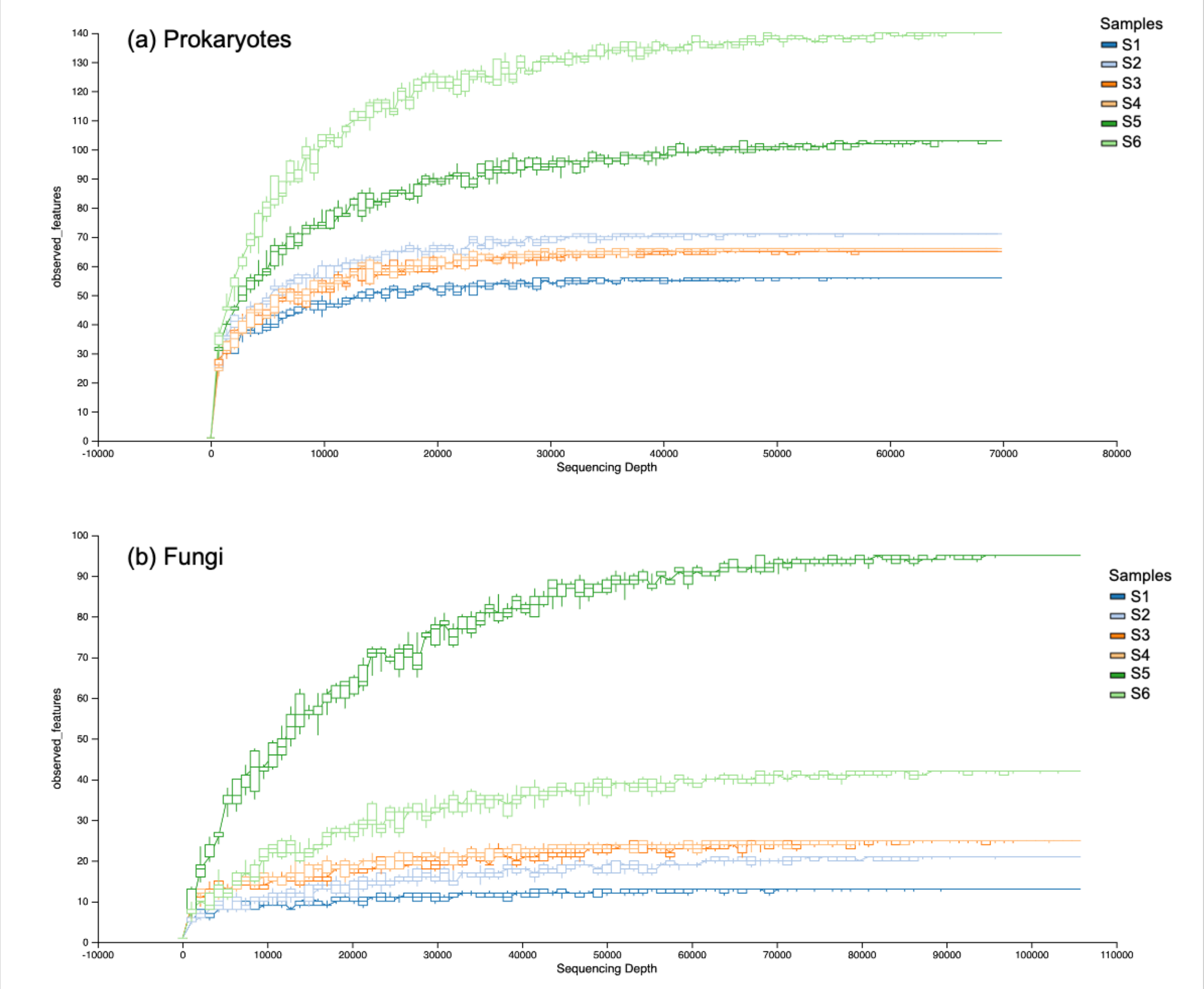

Supplementary Figure S1. Rarefaction curve showing observed feature in sample taken from the mural painting. (a) Prokaryotes and (b) fungi

Supplementary Table S2 Bacterial and fungal isolate and their NCBI accession number

Isolate	Accession number
Bacteria	
SKB1	ON527733
SKB2	ON527734
SKB3	ON527735
SKB4	ON527736
SKB5	ON527737
SKB6	ON527738
Fungi	
SKF1	ON532706
SKF2	ON532708
