# Supplementary material for: CIAO: a living experiment in interdisciplinary large-scale collaboration facilitated by the Adverse Outcome Pathway framework
Source: Front Public Health. 2023 Aug 10;11:1212544. doi: 10.3389/fpubh.2023.1212544 (PMC10449328; doi:10.3389/fpubh.2023.1212544)
Supplement: Supplementary file 2 [file Presentation_1.pdf]

Supplementary Material

CIAO: a living experiment in interdisciplinary large-scale collaboration facilitated by the Adverse Outcome Pathway framework

Annamaria Carusi, Julija Filipovska, Clemens Wittwehr and Laure-Alix Clerbaux*

*** Correspondence:** Laure-Alix Clerbaux, laure-alix.clerbaux@ec.europa.eu

1 Meta-level workshop: questions

We asked to the participants, for each of these questions, to try to think of concrete examples that came up during your interactions in the groups you were involved in.

The AOP Framework
Thinking about the core concepts of the AOP framework: KE, KER, MIE, and AO:
Was there agreement or consensus in your groups on the meaning of these concepts? Were they easily applied? Were there discussions about how they should be used or applied?
If there were disagreements or lack of consensus, were these resolved, and if so how?
Did your discussions around these terms add value to your collaboration, to knowledge and understanding? If so, how? If not, why not?

The AOP visuals
Was the diagram intuitive and easily understandable to you?
Did your group have discussions about the diagram?
If your group used the diagram, did they talk about changing it in any way? If so, how?
If you did not use the diagram, did you use anything else instead? If so, what was it?
Did the diagram add value to your collaboration / to knowledge and understanding? If so, how? If not, why not?

The AOP-Wiki
If your group used the Wiki, what did you use it for?
How intuitive was the Wiki? Did you find what you were looking for?
What was the best feature of the Wiki? What was the worst?
If you did not use the Wiki, what did you use for sourcing information about pathways ?
Did the Wiki add value to your collaboration and understanding. If so, how? If not, why not?

2 Interviews of some participants: questions

What is your background?
What brought you to CIAO?
Had you any previous experience working with the AOP framework? If not, how did you find it?
What was your experience of interdisciplinarity in CIAO?
Can you tell me about a particular experience of collaborating with someone from a different background on a CIAO activity?
